# Deep learning denoising reconstruction for improved image quality in fetal cardiac cine MRI

**DOI:** 10.3389/fcvm.2024.1323443

**Published:** 2024-02-12

**Authors:** Thomas M. Vollbrecht, Christopher Hart, Shuo Zhang, Christoph Katemann, Alois M. Sprinkart, Alexander Isaak, Ulrike Attenberger, Claus C. Pieper, Daniel Kuetting, Annegret Geipel, Brigitte Strizek, Julian A. Luetkens

**Affiliations:** ^1^ Department of Diagnostic and Interventional Radiology, University Hospital Bonn, Bonn, Germany; ^2^ Quantitative Imaging Lab Bonn (QILaB), Bonn, Germany; ^3^ Department of Pediatric Cardiology, University Hospital Bonn, Bonn, Germany; ^4^ Philips GmbH Market DACH, PD Clinical Science, Hamburg, Germany; ^5^ Department of Obstetrics and Prenatal Medicine, University Hospital Bonn, Bonn, Germany

**Keywords:** fetal imaging, Doppler ultrasound gating, congenital heart disease, deep learning, denoising, cardiac magnetic resonance imaging (CMR)

## Abstract

**Purpose:**

This study aims to evaluate deep learning (DL) denoising reconstructions for image quality improvement of Doppler ultrasound (DUS)-gated fetal cardiac MRI in congenital heart disease (CHD).

**Methods:**

Twenty-five fetuses with CHD (mean gestational age: 35 ± 1 weeks) underwent fetal cardiac MRI at 3T. Cine imaging was acquired using a balanced steady-state free precession (bSSFP) sequence with Doppler ultrasound gating. Images were reconstructed using both compressed sensing (bSSFP CS) and a pre-trained convolutional neural network trained for DL denoising (bSSFP DL). Images were compared qualitatively based on a 5-point Likert scale (from 1 = non-diagnostic to 5 = excellent) and quantitatively by calculating the apparent signal-to-noise ratio (aSNR) and contrast-to-noise ratio (aCNR). Diagnostic confidence was assessed for the atria, ventricles, foramen ovale, valves, great vessels, aortic arch, and pulmonary veins.

**Results:**

Fetal cardiac cine MRI was successful in 23 fetuses (92%), with two studies excluded due to extensive fetal motion. The image quality of bSSFP DL cine reconstructions was rated superior to standard bSSFP CS cine images in terms of contrast [3 (interquartile range: 2–4) vs. 5 (4–5), *P* < 0.001] and endocardial edge definition [3 (2–4) vs. 4 (4–5), *P* < 0.001], while the extent of artifacts was found to be comparable [4 (3–4.75) vs. 4 (3–4), *P* = 0.40]. bSSFP DL images had higher aSNR and aCNR compared with the bSSFP CS images (aSNR: 13.4 ± 6.9 vs. 8.3 ± 3.6, *P* < 0.001; aCNR: 26.6 ± 15.8 vs. 14.4 ± 6.8, *P* < 0.001). Diagnostic confidence of the bSSFP DL images was superior for the evaluation of cardiovascular structures (e.g., atria and ventricles: *P* = 0.003).

**Conclusion:**

DL image denoising provides superior quality for DUS-gated fetal cardiac cine imaging of CHD compared to standard CS image reconstruction.

## Introduction

Cardiac MRI is the preferred modality for imaging congenital heart disease (CHD) in children and adults, providing ventricular volumetry, functional analysis, and hemodynamic assessment without ionizing radiation (1). Novel technical advancements, such as Doppler ultrasound (DUS) gating, now enable the successful application of dynamic cardiac cine MRI to the fetus in clinical routine (2–6). In contrast to other fetal cardiac gating methods, such as metric-optimized gating and self-gating strategies, DUS allows for direct gating without the need for time-consuming image post-processing, thus enabling the application of clinically used standard sequences to the fetal heart with the ability to immediately review images and adjust planes, if necessary (7). Recently, DUS-gated cardiac cine imaging was demonstrated to detect pathologic findings in fetuses with CHD, even revealing cardiovascular malformations that were previously unknown (8–10). Furthermore, in inconclusive echocardiography, DUS-gated fetal cardiac MRI was found to aid clinical decision-making (11). Nevertheless, fetal cardiac MRI still poses challenges, and fetal cine images often suffer from a low signal-to-noise ratio (SNR) as a large field of view is needed relative to the small size of the fetal cardiovascular structures to avoid wrap-around artifacts caused by the maternal body (12). This is particularly challenging in obese pregnant women or in polyhydramnios, which leads to a further decrease in SNR (13). In addition, the rather low SNR is further decreased by the short acquisition times that are required to allow breath-holds despite limited maternal breathing capacity and also to minimize the influence of spontaneous fetal movement. Hence, for improved delineation of fetal cardiovascular structures, enhancing image contrast and reducing the amount of noise without lengthening acquisition time would be desirable.

Recently, deep learning (DL) image reconstruction based on convolutional neural networks (CNNs) has been demonstrated to improve image quality through noise reduction and to mitigate artifacts in various applications of clinical MRI, such as musculoskeletal or liver imaging (14–18). Even in other highly noise-corrupted imaging fields, e.g., in arterial spin labeling perfusion MRI or low-field MRI, DL models have provided substantial improvements in SNR and image quality (19, 20). Therefore, considering the specific challenges of fetal cardiac cine MRI, this study aimed to investigate an industry-developed DL reconstruction model that integrates a CNN for image denoising and to compare the DL-denoised cine images qualitatively and quantitatively with standard reconstructions, with special regard to image contrast, artifact level, and delineation of different fetal cardiovascular structures.

## Material and methods

### Study participants

This retrospective analysis of a prospectively enrolled study cohort was approved by the Institutional Review Board of the University Hospital Bonn. Pregnant women with CHD of the fetus as suspected by a second-trimester ultrasound scan were referred to the Department of Obstetrics and Prenatal Medicine of the University Hospital Bonn for further diagnostic workup and consecutively invited to undergo a fetal cardiac MRI from February 2022 to March 2023. Exclusion criteria were general contraindications for MRI. After the acquisition, data were checked and excluded from subsequent analysis as incomplete, e.g., due to strong fetal motion and therefore premature scan termination. All pregnant women gave their written informed consent to participate and for the publication of images prior to a fetal cardiac MRI examination.

### Fetal cardiac MRI acquisition

All fetuses were examined on a whole-body 3-T MRI system (Ingenia Elition X, Philips Healthcare). An anterior 28-channel torso coil and a posterior coil integrated into the patient table were used for signal reception. The specific absorption rate was operated in normal mode with a maximum limit of 2.0 W/kg. For retrospective gating, a DUS-based gating device was used as previously described (4). All participants were examined in a left lateral position to prevent vena cava compression and to increase maternal comfort. First, an MRI survey was scanned to plan the DUS transducer application for a correct alignment of the acoustic beam with the fetal heart. The imaging protocol consisted of standard T2-weighted single-shot turbo spin echo images in both the axial and sagittal planes oriented along the maternal craniocaudal axis to image the extra-cardiac thoracic anatomy, including the fetal lungs, and for further planning purposes. Multi-slice cine images in axial orientation were acquired as a consecutive stack using a DUS-gated 2D balanced steady-state free precession (bSSFP) sequence that was planned perpendicular to the fetal craniocaudal axis for dynamic visualization of the intra-cardiac anatomy and the thoracic vasculature. A compressed sensing (CS) acceleration factor of 3 was applied based on clinical experience prior to the study. The employed CS technique was based on a combination of compressed sensing and parallel imaging (Compressed SENSE, Philips Healthcare) (21). Other imaging parameters were as follows: field of view: 254 mm^2^ × 254 mm^2^; repetition time: 4.2 ms; echo time: 2.1 ms; flip angle: 65°; in-plane resolution: 1.72 mm^2^ × 1.45 mm^2^ (reconstructed: 0.99 mm^2^ × 0.99 mm^2^), slice thickness: 4 mm, no interslice gaps; breath-hold duration/slice: 8 s; temporal resolution: 17 ms; reconstructed cardiac phases: 25. Images were acquired during the maternal end-expiratory breath-hold.

### MRI data reconstruction

In addition to the conventional CS image reconstruction, an industry-developed DL algorithm was applied that was pre-trained and tested on approximately 740,000 sparsifying MR images of various anatomic areas and contrasts (SmartSpeed AI, Philips Healthcare). Initially described by Pezzotti et al., this network merges a CNN-based sparsification approach with the CS reconstruction approach (22). In doing so, the multi-scale sparsification from the wavelet transform of the conventional CS algorithm is replaced by a learned implementation, ensuring data consistency and incorporating domain-specific prior knowledge such as coil sensitivity distribution and location of the image background, as previously described (23). Trained for subsampling and reduction of related noise, the CNN is applicable to various MRI applications irrespective of the underlying anatomy and can therefore be used for fetal cardiac MRI, even though these images were not part of the training dataset. Unlike the initial model, which integrated a 2.5D multi-slice schema, the current network refrains from exploiting correlations between neighboring slices and the target slice to prevent errors arising from motion-induced variations in anatomical positions across individual slices. All images were reconstructed after the completion of data acquisition using the reconstruction hardware of the scanner, which is equipped with a dedicated graphics processing unit (NVIDIA RTX 5000). The reconstruction time per scan was less than 1 min.

### Quantitative MRI image analysis

Quantitative image analysis was performed by TV, a radiologist with 2 years of specific experience in DUS-gated fetal cardiac MRI and 3 years of experience in cardiac MRI of CHD (reader 1). Quantitative image quality assessment of the two reconstructions was conducted by calculating the apparent signal-to-noise ratio (aSNR) and apparent contrast-to-noise ratio (aCNR) based on the mean signal intensity and standard deviation within equal-sized regions of interest of the fetal left ventricular blood pool, fetal interventricular septal myocardium, and maternal abdominal wall muscle (rectus abdominis or abdominal oblique muscles) at end-diastole at the four-chamber view in the axial-oriented cine images. As previously described, aSNR was calculated by dividing the average signal intensity in the area of interest (myocardium) by the standard deviation of the background (muscle) (24, 25). aCNR was calculated as the difference between the mean signal intensity of the blood pool and the myocardium divided by the standard deviation of the muscle (26). To measure image sharpness, the edge rise distance was determined by analyzing the distance between the 10% and 90% signal intensity levels on a manually drawn line profile between the high-signal-intensity area of the left ventricular blood pool and the low-signal-intensity area of the septal myocardium. To allow an adjusted comparison, the line profile was interpolated to 10–3 mm using bicubic interpolation. Care was taken to ensure the exact alignment of the line profile perpendicular to the ventricular septal wall and consistent positioning in both reconstructions. For analysis of the edge rise distance, self-developed in-house software programmed in Matlab (Version R2022b, MathWorks) was used.

### Qualitative MRI image analysis

Subjective image quality was rated by reader 1 and JAL, a board-certified cardiovascular radiologist with 2 years of specific experience in DUS-gated fetal cardiac MRI and 11 years of experience in cardiac MRI of CHD (reader 2), for both the standard bSSFP CS and DL cine image stacks on a 5-point Likert scale in three categories: (1) blood-pool-to-myocardium contrast, (2) endocardial edge definition, and (3) level of artifacts, as previously described (27). Points were determined as follows:

1. Non-diagnostic: poor contrast between the blood pool and the myocardium; poorly defined endocardial edge; and artifacts render the images non-diagnostic.
2. Poor: the blood pool is barely discernible from the myocardium; a washed-out endocardial edge and blurred trabeculae are visible; and numerous artifacts are present.
3. Adequate: the blood pool is discernible from the myocardium but features lots of noticeable variations throughout the cardiac cycle; there is barely distinguishable endocardial edge definition; and some artifacts are present.
4. Good: the blood pool is mostly brighter and discernible from the myocardium; papillary and endocardial trabeculae are discernible but blurred in some images during the cardiac cycle; and a few artifacts are present but do not hinder image quality.
5. Excellent: the blood pool is hyperintense and clearly discernible from the myocardium in all images; papillary and endocardial trabeculae are clearly visible; and there are almost no artifacts.

For intra- and inter-reader reproducibility measurements, image quality assessment was performed independently by the two readers and was repeated by the first reader four weeks after the initial rating. For analysis, the reconstructed image datasets were blinded and randomized. The window levels of the images were preset the same for both readers but could be adjusted by each reader during the evaluation process. Ratings of both readers are given in the manuscript.

Diagnostic confidence for the evaluation of the atria, ventricles, foramen ovale, atrioventricular and semi-lunatic valves, great thoracic vessels, aortic arch, and pulmonary veins was analogously assessed with grade 1 if there was no confidence in the validity of the image stack; grade 2 if there was poor confidence; grade 3 if there was moderate confidence; grade 4 if there was good confidence; and grade 5 if there was high confidence due to excellent delineation of the structure. Diagnostic confidence assessment was performed in a consensus reading fashion by the two readers.

### Statistical analysis

Prism (Version 9.5.1; GraphPad Software) and SPSS (Version 27, IBM Corp.) were used for statistical analysis. Dichotomous variables are summarized as percents of absolute frequency. Continuous variables are given as mean ± standard deviation, and discrete variables are presented as median and interquartile range. Image quality and diagnostic confidence scores for both reconstructions were compared using the Wilcoxon test. Intra- and inter-observer reproducibility on grades of image quality was assessed using intraclass coefficient (ICC) estimates (<0.5: poor, 0.5–0.75: moderate; 0.75–0.9: good, >0.9: excellent). Intraclass coefficient estimates and their 95% confidence intervals (CIs) were based on a single-measure, two-way mixed (consistency) model. The level of statistical significance was set to *P* < 0.05.

## Results

**Figure 1:**
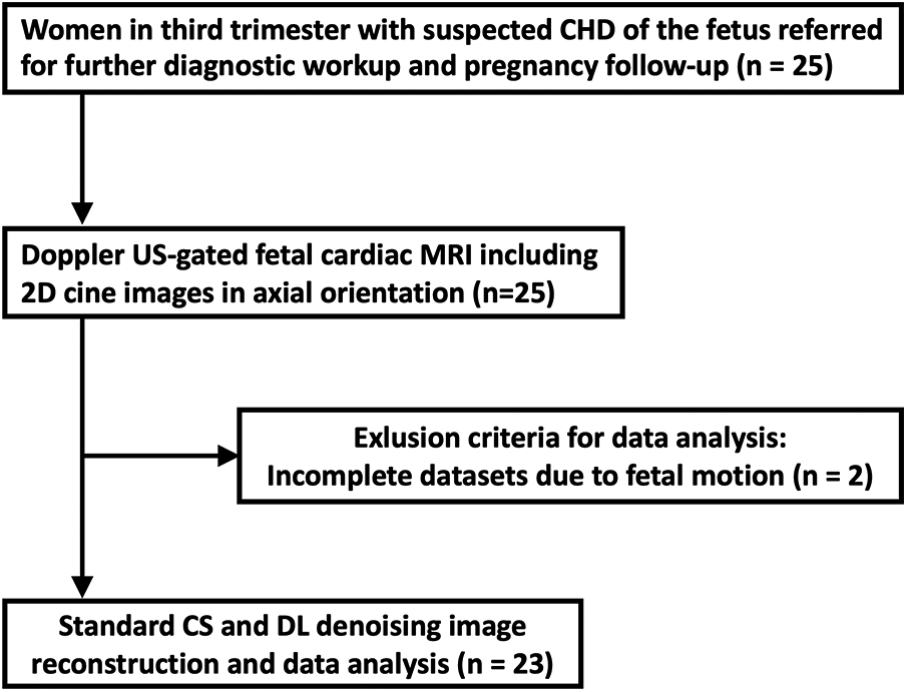
Study flow diagram.

Twenty-five pregnant women were included and underwent DUS-gated fetal cardiac MRI without fetal sedation. Of these, two were excluded from further analysis because of extensive fetal bulk movements during the MRI scan, leading to an unstable gating signal and therefore premature scan termination. A total of 23 women (mean age: 32 ± 4 years) at a mean gestational age of 35 ± 1 weeks completed the examination and were consecutively included for further data reconstruction and analysis (Figure 1). Underlying CHD of the fetuses included univentricular heart defects (7 of 23, 30%), aortic and pulmonary valve diseases (5 of 23, 22%), aortic arch malformations (3 of 23, 13%), and other conditions (9 of 23, 39%). Detailed participant and fetal characteristics are given in Table 1.

**Table 1:** Clinical characteristics of the study collective.

Parameters	Value
Participants characteristics	
Participants	23
Maternal age at MRI (years)	32 ± 4^a^
Gestational age at MRI (weeks)	35 ± 1^a^
Congenital heart disease of the fetus	
Univentricular heart	7 (30)
HLHS	5 (23)
DILV	1 (4)
DORV	1 (4)
Valve disease	5 (22)
Pulmonary valve atresia/stenosis	3 (13)
Aortic valve stenosis (excluding HLHS)	2 (9)
Aortic arch disease	3 (13)
Interrupted aortic arch	1 (4)
Double aortic arch	1 (4)
Right aortic arch	1 (4)
Others (AVSD, d-TGA, cc-TGA, diverticulum)	9 (39)

Unless otherwise indicated, data are the number of fetuses with percentages in parentheses.

AVSD, atrioventricular septal defect; cc-TGA, congenitally corrected transposition of the great arteries; DILV, double-inlet left ventricle; DORV, double-outlet right ventricle; d-TGA, dextro-transposition of the great arteries; HLHS; hypoplastic left heart syndrome.

^a^ Data are presented as mean ± standard deviation.

### Quantitative comparison

**Figure 2:**
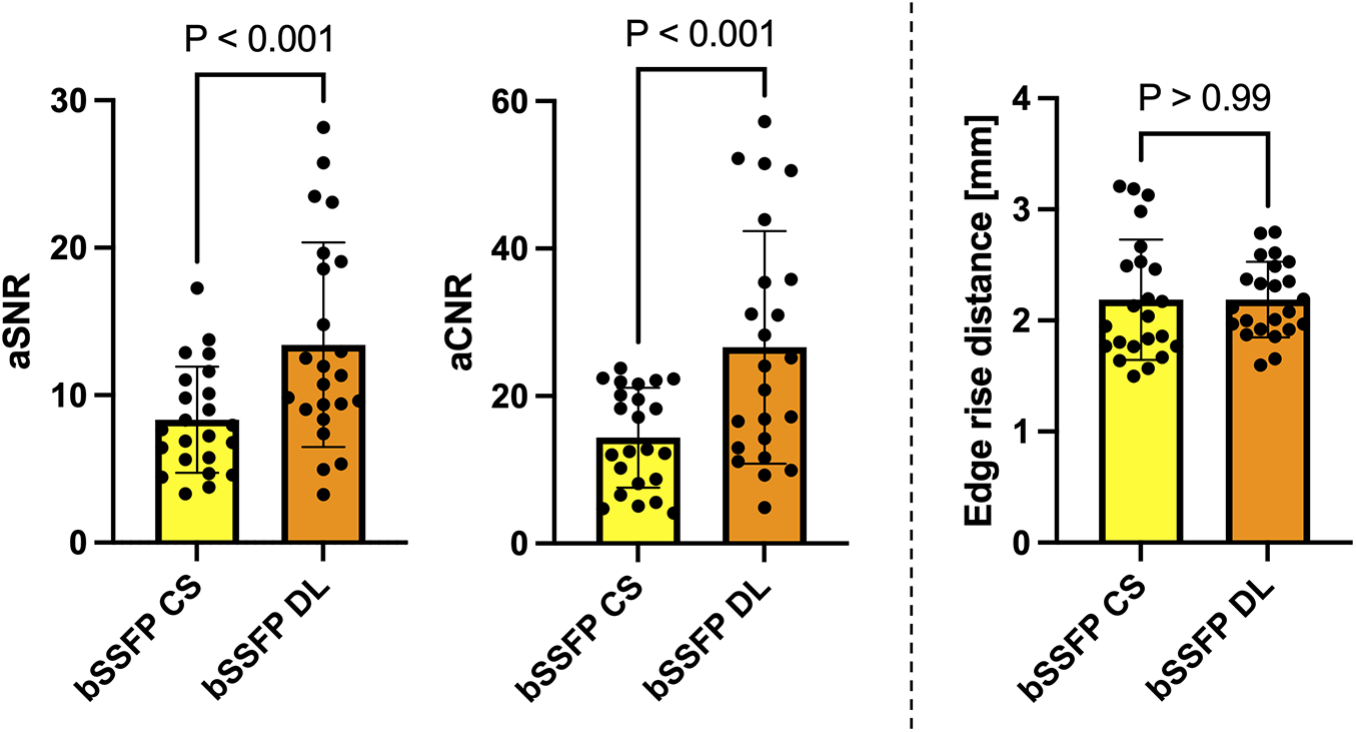
Column graphs with individual plotted values showing quantitative analysis metrics for each fetus stratified by reconstruction type. Distributions are given for the aSNR, aCNR, and edge rise distance. Data are mean with standard deviation error bars.

The bSSFP DL cine had a significantly higher aSNR than the standard bSSFP CS cine (13.4 ± 6.9 vs. 8.3 ± 3.6, *P* < 0.001) (Figure 2). Similarly, the aCNR of the bSSFP DL cine was significantly higher than that of the standard bSSFP CS cine (26.6 ± 15.8 vs. 14.4 ± 6.8, *P* < 0.001). The edge rise distance as a measure for the image sharpness revealed no difference between the two reconstruction frameworks (2.2 mm ± 0.3 vs. 2.2 mm ± 0.5, *P* > 0.99).

### Qualitative comparison

**Figure 3:**
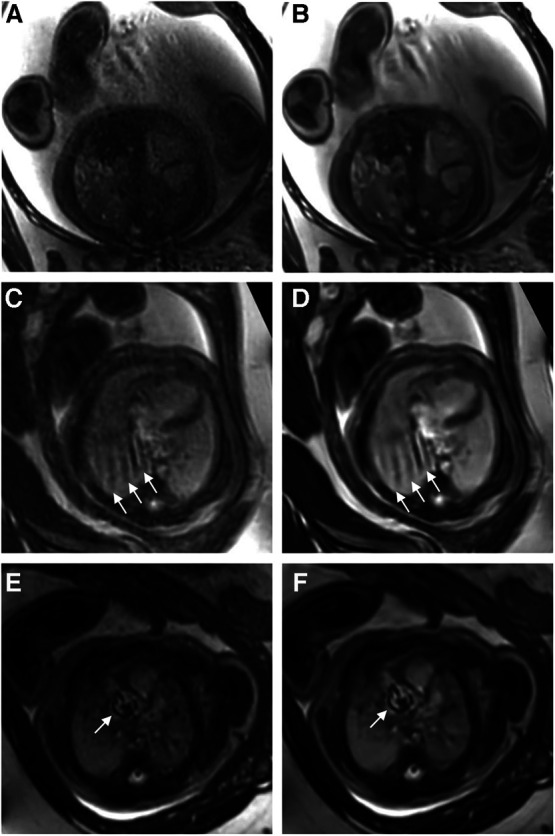
(**A**,**C**,**E**) Standard CS and corresponding (**B**,**D**,**F**) DL image reconstructions of an axial balanced steady-state free precession cine sequence affected by different types of artifacts, including dielectric effects (**A**,**B**), maternal breathing (arrows in **C**,**D**), and arterial pulsation (arrows in **E** and **F**). No attenuation of artifacts was noticed by using the DL denoising algorithm for image reconstruction compared to the standard images. In fact, the pulsation artifacts in (**F**) caused by aortic valve stenosis seem even more pronounced compared to the corresponding standard image (**E**); the complete axial cine imaging stacks of both the standard CS reconstructions (Supplementary Movie S1) and the DL reconstructions (Supplementary Movie S2) are provided.

Reader 1 rated the bSSFP DL cine as significantly better for image contrast [3 (2–4) vs. 5 (4–5), *P* < 0.001] and endocardial edge definition [3 (2–4) vs. 4 (4–5), *P* < 0.001]. Regarding artifacts, CS and DL reconstructions scored equally [4 (3–4.75) vs. 4 (3–4), *P* = 0.4] (see Table 2 for quality ratings by reader 2). Intra- and inter-observer reproducibility was good or excellent for rating image contrast and endocardial edge definition, whereas the intraclass correlation coefficient was moderate or good for reproducibility regarding artifacts (see Table 3). Minor artifacts occurred in all 23 fetuses (e.g., motion artifacts), which did not hinder the diagnostic assessment of cardiovascular structures. New artifacts related to the DL reconstruction were not noted. Examples of image artifacts are provided in Figure 3.

**Table 2:** Quality ratings by readers 1 and 2.

	Category	bSSFP CS^a^	bSSFP DL^a^	*P-*value
Reader 1	Contrast	3 (2–4)	5 (4–5)	**<0** **.** **001**
	Endocardial edge definition	3 (2–4)	4 (4–5)	**<0**.**001**
	Artifacts	4 (3–4.75)	4 (3–4)	0.40
Reader 2	Contrast	3 (2.25–4)	4.5 (4–5)	**<0**.**001**
	Endocardial edge definition	3.5 (3–4)	4 (3–5)	**0**.**003**
	Artifacts	3 (2.25–4)	3 (2–3)	0.18

Bold indicates statistical significance.

^a^ Data are presented as median with interquartile range in parentheses.

**Table 3:** Intra- and inter-observer reproducibility for image quality ratings by readers 1 and 2.

	Category	ICC (95% CI) bSSFP CS	ICC (95% CI) bSSFP DL
Intra-observer reproducibility	Contrast	0.87 (0.68–0.94)	0.86 (0.67–0.94)
	Endocardial edge definition	0.91 (0.79–0.96)	0.90 (0.77–0.96)
	Artifacts	0.74 (0.39–0.89)	0.78 (0.43–0.90)
Inter-observer reproducibility	Contrast	0.78 (0.48–0.91)	0.84 (0.63–0.93)
	Endocardial edge definition	0.73 (0.36–0.89)	0.76 (0.44–0.90)
	Artifacts	0.71 (0.32–0.88)	0.76 (0.43–0.090)

### Comparison of diagnostic confidence

**Figure 4:**
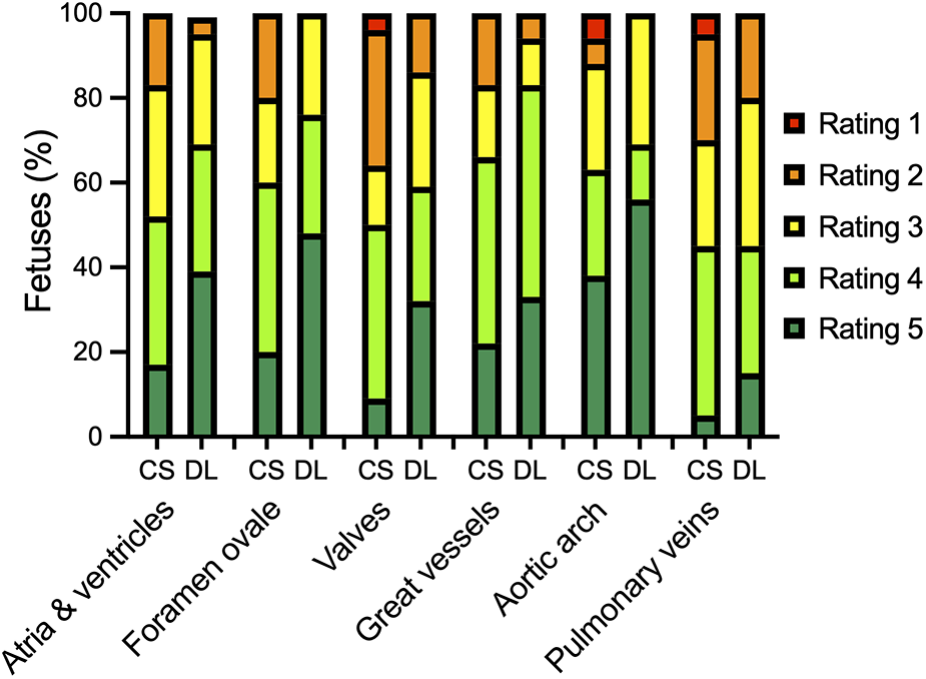
Bar plot showing the distribution of ratings for diagnostic confidence in assessing different cardiovascular structures using standard CS and DL reconstructions of a balanced steady-state free precession cine sequence. The 5-point Likert scale ratings were defined as follows: grade 1, if there was no confidence in the validity of the image stack; grade 2, if there was poor confidence; grade 3, if there was moderate confidence; grade 4, if there was good confidence; and grade 5, if there was high confidence due to excellent delineation of the structure.

**Figure 5:**
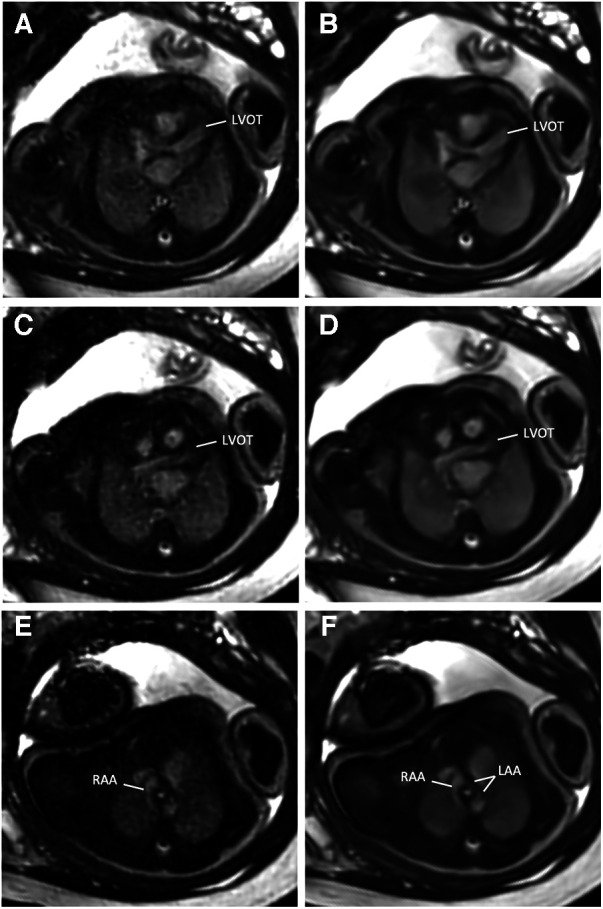
(**A**,**C**,**E**) Standard CS and corresponding (**B**,**D**,**F**) DL image reconstructions of an axial balanced steady-state free precession cine sequence in a 38-year-old woman at a gestational age of 35 weeks and 2 days with a double aortic arch of the fetus. DL denoised images provided better delineation and diagnostic confidence of cardiovascular structures from the left ventricular outflow tract (LVOT, **A**–**D**) to the aortic arch level (**E**,**F**). While the dominant right-sided aortic arch (RAA) can be clearly delineated with both reconstruction models (**E**,**F**), the narrow left-sided aortic arch (LAA) is more clearly visible in the DL denoised image (**F**).

**Figure 6:**
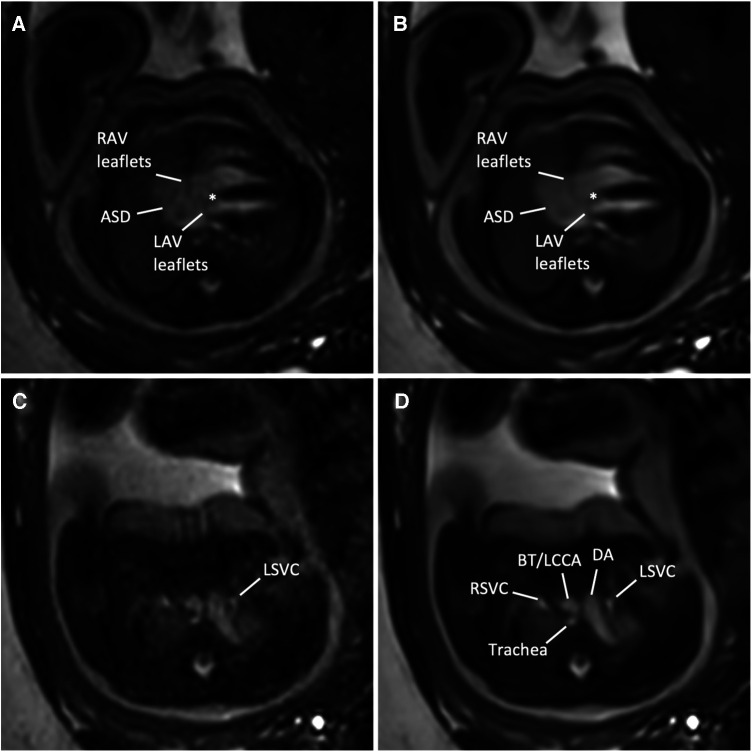
(**A**,**C**) Standard CS and corresponding (**B**,**D**) DL image reconstructions of an axial balanced steady-state free precession cine sequence in a 35-year-old woman at a gestational age of 36 weeks and 3 days with an intermediate atrioventricular (AV) septal defect of the fetus. Images at AV level (**A**,**B**) revealed a large (type I and II) atrial septal defect (ASD), right AV and left AV leaflets, and a small defect in the basal portion of the interventricular septum (asterisk), indicating an intermediate-type AV canal. Note the clear delineation of the AV leaflets in the DL denoised image (**B**) compared with the standard CS reconstructed image (**A**) In addition, MRI revealed a left persistent superior vena cava (LSVC, **C**,**D**), draining into the coronary sinus (not shown). Adjacent structures at this level are the right superior vena cava, the ductus arteriosus, the supra-aortic vessels, including the brachiocephalic trunk (BT) and the left common carotid artery (LCCA), and the trachea. Both standard CS (Supplementary Movie S3) and DL-reconstructed cine images at the AV level (Supplementary Movie S4) are also provided. DA, ductus arteriosus; LAV, left atrioventricular valve orifice; RAV, right atrioventricular valve; RSVC, right superior vena cava.

Confidence in diagnostic reading was rated higher when the bSSFP DL cine images were used to assess different cardiovascular structures (see Figure 4 and Table 4). Image examples demonstrating higher diagnostic confidence of bSSFP DL images for evaluation of the atrioventricular valves in a fetus with an intermediate-type atrioventricular septal defect and evaluation of the double aortic arch in another fetus are provided in Figures 5, 6.

**Table 4:** Diagnostic confidence of different cardiovascular structures for both balanced steady-state free precession cine CS and DL reconstruction models, as evaluated by consensus reading.

Structure	bSSFP CS^a^	bSSFP DL^a^	*P*-value
Atria and ventricles	4 (3–4)	4 (3–5)	**0** **.** **003**
Foramen ovale	4 (3–4)	4 (3.5–5)	**0** **.** **001**
Atrioventricular and semi-lunatic valves	3.5 (2–4)	4 (3–5)	**<0** **.** **001**
Great thoracic vessels	4 (3–4)	4 (4–5)	**0** **.** **016**
Aortic arch	4 (3–5)	5 (3–5)	**0** **.** **031**
Pulmonary veins	3 (2–4)	3 (3–4)	**0** **.** **031**

Bold indicates statistical significance.

^a^ Data are presented as median with interquartile range in parentheses.

## Discussion

This study investigated an industry-developed DL denoising reconstruction for image quality improvement of fetal cardiac cine MRI in different forms of CHD. DL cine images provided higher aSNR and aCNR and improved diagnostic confidence for the assessment of cardiovascular structures in comparison with standard CS reconstruction.

As occurred in two studies in this work, arbitrary fetal motion is common and may lead to premature termination of data acquisition due to DUS gating signal loss. Although methods for motion compensation are evolving and might be vendor-provided in the future (28–32), repetition of incomplete sequences is often needed to achieve diagnostic information. As most cine sequences are being performed during breath-hold, multiple repetitions may deplete breathing capacity and comfort, leading to insufficient image quality. Therefore, acceleration of data acquisition by using CS is particularly beneficial for fetal cardiac cine MRI (31, 33). Yet, this advantage is partially offset by increased apparent image noise due to the incoherency of data undersampling when standard reconstruction methods are used (34, 35).

The DL model used in this study has been proven to alleviate the limitations of CS techniques in knee and prostate MRI and in MR cholangiopancreaticography and has received 510(k) clearance from the U.S. Food and Drug Administration (22, 23, 36, 37). Therefore, the purpose of this study was to evaluate the performance of the technique for feasible and easy-to-implement image denoising of fetal cardiac cine images in a clinical setting of fetuses with various forms of CHD. Quantitative analysis revealed a higher apparent signal-to-noise ratio and contrast-to-noise ratio of the DL cine reconstructions than the standard CS cine reconstructions (*P* = 0.002 and *P* < 0.001, respectively). In addition, qualitative reader assessment indicated that the DL cine reconstructions improved image quality, particularly in terms of endocardial edge definition and contrast (*P* < 0.001). These results are in concordance with previously reported measurements in prostatic lesions and intrahepatic bile ducts (23, 36). Furthermore, the level of diagnostic confidence was increased for six different cardiovascular structures, including the cardiac chambers, foramen ovale, valves, great thoracic vessels, aortic arch, and pulmonary veins. This could be of particular relevance for a precise analysis of ventricular morphology, size, and function to assess univentricular vs. biventricular outcomes in the borderline left ventricle (11). Another possible diagnostic benefit of better depiction of the foramen ovale might exist in hypoplastic left heart syndrome or dextro-transposition of the great arteries with unclear interatrial communication due to poor acoustic windows in late gestation, potentially leading to changes in delivery mode planning with catheterization laboratories on standby or not (11). Although the level of significance was weaker compared to the other structures (*P* = 0.031), the delineation of the aortic arch and the pulmonary veins could also be improved by using the DL cine reconstructions for diagnostic reading. This would be advantageous, for example, for better visualization of anomalous pulmonary venous connections or for the evaluation of aortic isthmus stenosis, which is considered one of the most underdiagnosed conditions in prenatal echocardiography (38). Although no aortic isthmus stenosis was present in this study sample to verify this, in one fetus with a double aortic arch, the smaller left-sided aortic arch could be more clearly delineated using the DL reconstructions compared to the standard CS reconstruction (Figure 5). However, to gain additional diagnostic information on extra-cardiac vessels alongside fetal echocardiography, it is worth noting that the inclusion of phase-contrast flow measurements and 3D volume datasets may be beneficial compared to 2D cine imaging alone.

While DL denoising reconstructions can improve image quality for various sequences within short reconstruction times (<1 min), certain limitations persist. DL images do not mitigate artifacts arising from fetal movements, maternal breathing, or arterial pulsation. Fetal movements pose a significant challenge for the diagnostic quality of fetal MRI, necessitating additional motion compensation techniques. Notably, we found that artifacts become even more pronounced in some DL-reconstructed images, possibly attributed to increased image contrast, as noticeable, for example, in pulsation artifacts. While this may hold diagnostic value as an indicator of aortic or pulmonary valve stenosis (Figure 3), accentuation of noise-related artifacts may also lead to misinterpretation and false-positive findings.

Despite a better endocardial edge definition revealed by the qualitative image assessment, we found no difference in the edge rise distance between the myocardium and the blood pool as a quantifiable surrogate for the image sharpness. This can be explained by the fact that in this DL model, applied prior to coil combination, the CNN removes noise from the images but does not replace the traditional zero-filling strategy to increase the matrix size. However, this can be realized by applying a series of CNNs for DL-based super-resolution reconstruction (39). The application of DL super-resolution to cardiac cine imaging in healthy fetuses has been demonstrated to result in faster data acquisition while maintaining image quality (40). However, to what extent these advanced reconstruction pipelines lead to better detection of pathologic structures and a higher success rate for fetal cardiac MRI in the clinic is a matter of future research.

A limitation of this single-center study is the small number of fetuses included, despite a wide range of underlying CHD. Due to this heterogeneity, diagnostic performance could not be elicited for individual CHD subtypes. Second, the commercially available DL reconstruction method used in this study was not specifically developed for fetal cardiac imaging. However, DL models for adult image data may be less effective in recovering the large-scale field of view and the detailed dynamic properties of fetal cardiac MRI, suggesting more targeted training and evaluation (41). Moreover, although a thorough investigation of different acceleration factors related to the effect of the DL reconstruction may be useful, we fixed and applied a moderate factor of 3 to a clinically used bSSFP CS sequence to keep the scan time reasonable in the clinical setup based on initial experience prior to the study. Furthermore, we solely focused on anatomic imaging for structural evaluation and did not include flow measurements for hemodynamic assessment, which can be part of a comprehensive fetal cardiac MRI scan in CHD. As the underlying principles of DL denoising can be applied to various sequences, further evaluation in phase-contrast flow MRI is warranted. Another potential application could involve the denoising of real-time cine images if no gating method is available. Finally, despite efforts being made to ensure image quality assessment in a blinded fashion, distinctive differences between standard bSSFP CS and DL cine reconstructions were obvious to the readers and thus introduced potential bias.

In conclusion, our study results demonstrate that DL denoising reconstruction improves the image quality of fetal cardiac cine MRI. It is essential to note, however, that image artifacts can be more pronounced when using DL reconstructions, which might introduce a risk of misinterpretation. Still, compared with the standard CS reconstruction, DL images enable higher diagnostic confidence for structural assessment in fetuses with different forms of CHD.

## Data Availability

The original contributions presented in the study are included in the article/Supplementary Material, further inquiries can be directed to the corresponding author.
